# Assessment of miscarriage factors among Latinas who live in the U.S.: a cross-sectional study

**DOI:** 10.3389/fgwh.2023.1127695

**Published:** 2023-04-26

**Authors:** Madeline Fernandez-Pineda, Brian E. McCabe, Rosina Cianelli, Natalia Villegas, Lilian Ferrer, Nilda Peragallo Montano

**Affiliations:** ^1^ Department of Nursing Science, College of Nursing, East Carolina University, Greenville, NC, United States; ^2^ Department of Special Education, Rehabilitation, and Counseling (SERC), College of Education, Auburn University, Auburn, AL, United States; ^3^ School of Nursing and Health Studies, University of Miami, Coral Gables, FL, United States; ^4^ Pontificia Universidad Catolica de Chile, Escuela de Enfermeria, Santiago, Chile; ^5^ School of Nursing, University of North Carolina at Chapel Hill, Chapel Hill, NC, United States

**Keywords:** Hispanic/Latinas, miscarriage, pregnancy loss, acculturation, intimate partner violence, sociodemographic, midwifery care

## Abstract

**Introduction:**

Latinas in the US are underrepresented in miscarriage research, yet face several risk factors for having a miscarriage, including intimate partner violence, and increasing maternal age. Increased acculturation is associated to increased risk of intimate partner violence and adverse pregnancy outcomes among Latinas yet is also understudied in the realm of miscarriage. Thus, this study aimed to analyze and compare sociodemographic characteristics, health-related factors, intimate partner violence, and acculturation among Latinas with and without a history of miscarriage.

**Methods:**

This study utilizes a cross-sectional design to analyze baseline data from a randomized clinical trial on the effectiveness of “Salud/Health, Educación/Education, Promoción/Promotion, y/and Autocuidado/Self-care” (SEPA), a human immunodeficiency virus risk reduction intervention for Latinas. Survey interviews were conducted in a private room at the University of Miami Hospital. Survey data analyzed include demographics, a bi-dimensional acculturation scale, a health and sexual health survey, and the hurt, insult, threaten, and scream tool. This study's sample was 296 Latinas, 18 to 50 years old, with and without a history of miscarriage. Data analyses included descriptive statistics, *t*-tests for continuous variables, negative binomial for counts, and chi-square for dichotomous or categorical variables.

**Results:**

Most Latinas were Cuban (53%), lived in the U.S. an average of 8.4 years, had 13.7 years of education, and a monthly family income of $1,683.56. Latinas with history of miscarriage were significantly older, had more children, more pregnancies, and poorer self-rated health than Latinas without history of miscarriage. Although not significant, a high percentage of intimate partner violence (40%) and low levels of acculturation were reported.

**Discussion:**

This study contributes new data about different characteristics of Latinas who have and have not experienced a miscarriage. Results can help identify Latinas at risk for miscarriage or its adverse-related outcomes and help develop public health policies focusing on preventing and managing miscarriage among Latinas. Further research is warranted to determine the role of intimate partner violence, acculturation, and self-rated health perceptions among Latinas who experience miscarriage. Certified nurse midwives are encouraged to provide Latinas with culturally tailored education on the importance of early prenatal care for optimal pregnancy outcomes.

## Introduction

In the United States (U.S.), an estimated 30%–40% of all pregnancies and 15%–20% of clinically recognized pregnancies end in *miscarriage*, or the loss of a pregnancy before 20 weeks gestation (1, 2). It is estimated that miscarriage is the most common form of pregnancy loss, with 80% of early pregnancy loss occurring in the first trimester of gestation (3). The risk is multivariate and while there are risk factors (e.g., maternal age, prior obstetrical history, and maternal comorbidities), it is difficult to establish predictors of future pregnancy loss (3).

Latinas, a vulnerable and marginalized population, are often underrepresented in research studies on women who experience miscarriage. Yet, U.S. Latinas face several risk factors for

having a miscarriage when compared to non-Latina White women, including, for example, more severe phenotypes of polycystic ovarian syndrome (4), and higher cases of Chlamydia [524.2 vs. 274.7 per 100,000; (5)] and Gonorrhea infections [88 vs. 65.5 per 100,000; (6)]. Additionally, age of first pregnancy has increased from 2000 to 2014 among Latinas of Cuban (26.5 to 27 years) and Central and South American descent (24.8 to 26.5 years) and are comparable to the age of first pregnancy among non-Latina Whites [which increased from 25.9 to 27 years; (7)]. Most miscarriages (about 60%) occur randomly due to a genetic problem which becomes even more common in women of increased reproductive age (8).

Several studies have also identified an association between miscarriage and intimate partner violence [IPV; (9, 10)], an issue that is quite prevalent in the Latino community (11, 12). Immigration and acculturation to a new culture have been cited as risk factors for IPV due to changes in existing gender roles within Latino families. Acculturation has also been associated with increases in alcohol abuse, unemployment, and socioeconomic issues all of which predict IPV (13). Furthermore, higher levels of acculturation have been associated to increased risk of IPV and adverse pregnancy outcomes including preterm births, preeclampsia or eclampsia, and gestational hypertension (13, 14). Nonetheless, IPV and acculturation have been scarcely studied among Latinas within the realm of miscarriage. Limited data on Latinas who have experienced a miscarriage means that certified nurse midwives have little evidence to guide and tailor their practice to provide adequate care to these women. Thus, this study aimed to analyze and compare sociodemographic characteristics, health-related factors, intimate partner violence, and acculturation among Latina women with and without a history of miscarriage.

## Materials and methods

### Design

This study uses a descriptive cross-sectional study design to examine the variables of interest with baseline data of a larger randomized trial on the effectiveness of Salud/Health, Educación/Education, Promoción/Promotion, y/and Autocuidado/Self-care(SEPA), a culturally specific and theoretically based group intervention for Latinas (15). The Florida Department of Health Institutional Review Board approved this study.

### Setting and participants

Eligibility criteria for the parent study included participants being 18 to 50 years old, self-identifying as a Latina, and sexually active within six months prior to study participation. Participants were recruited using convenience and snowball sampling from the Miami Refugee Center, Florida Department of Health, and public locations, including grocery stores, churches, and community organizations. Of women assessed for eligibility, the parent study had a response rate of 59%. After participants read and signed the informed consent form, the survey interviews were conducted in a private room at the University of Miami Hospital. A total of 320 surveys were conducted by trained bilingual female researchers in the participants’ preferred language (Spanish or English) utilizing a standardized protocol on a secure web-based software (e-Velos). Of those 320, a sub-sample of 296 Latinas provided information on their miscarriage history, making up the sample for this study (*n =* 89 who reported experiencing at least one miscarriage and *n =* 207 who did not have any miscarriage).

### Measures

Participant sociodemographic characteristics were collected using an 18-item standardized demographic intake form (15). Eleven items, age, years living in the U.S., preferred language, monthly family income, relationship status, educational level, employment status, health insurance status, religious service attendance, religious identity, and religious strength influencing life were examined for this study.

Acculturation was assessed with the bidimensional acculturation scale [BAS; (16)] to measure participant's acculturation level. It consists of 24 items to assess Latino's level of acculturation to the U.S. culture (*Americanism; 12 items*) and to their culture of origin (*Hispanicism; 12 items*). Items are based on language proficiency and frequency of use for speaking, reading, and media consumption. The range of scores for each cultural dimension is 1–4. A score of 2.5 or higher either dimension (i.e., Hispanic or U.S.) indicates a high level of acculturation to that dimension. At baseline in this sample, both the Hispanicism and Americanism subscales had high internal consistency (*α* = .82 and.96, respectively).

Health-related factors, such as the number of pregnancies, miscarriages, and live children in addition to if they had a regular healthcare provider, their perceived health status, and history of sexually transmitted infections (STIs) were assessed with items from the health and sexual health 8-item survey (17). Self-rated health status used a 4-point Likert (i.e., very good, good, poor, very poor) and was coded as *good* if participants chose either “Very good” or “good” answer choices and *poor* if they chose “Very poor” or “poor” answer choices. History of STIs was coded as “yes” if any were reported and “no” if none were reported.

Intimate partner violence (IPV) in the last month was assessed using the hurt, insult, threaten, and scream [HITS; (18)] tool which measures four types of IPV. Response choices are on a 5-point Likert scale (1 = never, 2 = rarely, 3 = sometimes, 4 = fairly often, and 5 = frequently). IPV responses were counted as “yes” if participants reported any type of IPV (i.e., options 2–5). The tool had a *α* = .91 of internal consistency. (see Supplementary Appendix for all measures used in this study).

### Statistical analysis

IBM SPSS 27 was used for all analyses. Sociodemographic characteristics shown with descriptive statistics. Then, we tested whether several variables were different between Latinas with or without a miscarriage using *t*-tests for continuous variables, negative binomial for counts, and chi-square (*χ*²) for dichotomous or categorical variables. Table 1 shows Spearman rank correlations between all study variables.

**Table 1:** Spearman rank correlations between study variable*s.*

Variables	1	2	3	4	5	6	7	8	9	10	11	12	13	14	15	16	17	18	19	20	21
1. Age, years	1																				
2. Years in US	.13*	1																			
3. Education	.20**	−.24**	1																		
4. Family Income	.13*	.22**	.12*	1																	
5. Hispanicism	.07	−.23**	.11*	.06	1																
6. Americanism	−.33**	.39**	.11	.14*	−.32**	1															
7. Spanish preferred	.24**	−.34**	.10	.02	.29**	−.41**	1														
8. Spouse/Partner	.08	−.07	.06	.10	−.09	−.17**	.01	1													
9. Employed	.01	.19**	.01	.16**	−.08	.18**	−.02	−.03	1												
10. Weekly Religious	.03	.06	−.04	−.03	.12*	.06	.13*	.08	.01	1											
11. Very Religious	.10	.05	−.03	.05	.09	−.01	.04	.02	.07	.26**	1										
12. Strong Religious	.10	.05	−.03	.05	.09	−.01	.04	.02	.07	.26**	1.00**	1									
13. Health Insurance	−.06	−.20**	.11	.03	.04	.01	−.03	−.11	−.11*	−.08	−.06	−.06	1								
14. # Children	.34**	.28**	−.21**	.13*	.04	−.14*	.06	.10	.03	.20**	.10	.10	−.02	1							
15. # Pregnancies	.44**	.08	−.07	.04	.02	−.24**	.10	.07	−.04	.06	.02	.02	.08	.73**	1						
16. Good Health	.01	−.13*	.03	.13*	.02	−.05	.16**	.02	−.05	.01	.03	.03	.06	−.08	−.18**	1					
17. Regular Provider	−.04	−.05	.03	.05	.00	.12*	−.08	−.02	−.12*	.02	−.01	−.01	.58**	.05	.11*	−.04	1				
18. Physically Hurt	−.04	.07	−.15**	.00	−.12*	−.03	−.01	−.10	.01	−.01	.00	.00	−.09	.01	.07	.04	−.04	1			
19. Insulted	.04	.16**	−.11	.08	−.01	.06	−.16**	−.04	−.01	−.01	.04	.04	−.06	.06	.05	−.05	.01	.31**	1		
20. Threatened	.01	.04	−.08	.04	.00	−.07	.01	−.07	−.02	.00	.04	.04	−.06	.06	.06	.02	−.03	.61**	.41**	1	
21. Screamed at	−.02	.24**	−.12*	.15**	−.05	.11*	−.24**	.01	.03	−.01	−.04	−.04	−.08	.09	.04	.02	.02	.46**	.65**	.45**	1

* *p* < .05.

** *p* < .001.

## Results

### Sociodemographic characteristics

Most Latinas in this study were born in Cuba (53%), followed by Nicaragua (10%), Honduras (9%), and Colombia (9%). Of the remaining 19%, no nationality had more than 5% and included the Dominican Republic, the U.S., Venezuela, Peru, Guatemala, El Salvador, Mexico, Panama, Argentina, Puerto Rico, Ecuador, and Bolivia. Latinas with a history of miscarriage made up 30% of the sample, of which 82% had one and 18% had two to six miscarriages. Latinas who experienced a miscarriage were significantly older (*M *= 38.82, *SD *= 7.92) t = 5.01, *p* < .001 than Latinas with no history of miscarriage (*M *= 33.21, *SD *= 9.19). See Table 2 for all sample sociodemographic data and group comparisons.

**Table 2:** Sociodemographic Characteristics of Latinas with a History of Miscarriage.

Characteristics	No Miscarriage (*n* = 207)		Miscarriage (*n* = 89)		*t*	*p*
	*M*	*SD*	*M*	*SD*		
Age, years	33.21	9.19	38.82	7.92	5.01	<.001
Years in US	8.41	8.14	8.71	8.92	0.28	.776
Education, years	13.75	3.21	13.98	4.00	0.51	.611
Monthly Family Income, USD	1,683.56	1,043.45	1,661.17	911.76	0.18	.861
Hispanicism	3.47	0.38	3.50	0.35	0.61	.545
Americanism	2.07	0.80	1.88	0.73	1.93	.055
	*n*	%	*n*	%	*χ*²/*b*	*p*
Spanish preferred	192	93	85	96	0.79	.449
Has Spouse/Partner	158	76	63	71	1.01	.382
Employed	61	29	23	26	0.40	.576
Weekly Religious Service Attendance	51	25	23	26+	0.05	.826
Very Religious Identity	17	8	9	10	0.28	.596
Very Strong Religious Strength Influencing Life	59	29	27	30	0.12	.725
Has Health Insurance	95	46	39	44	0.11	.742

### Acculturation

Women with a history of miscarriage scored less than 2.5 in the Americanism (*M *= 1.88, *SD *= 0.73) component of BAS and more than 2.5 in the Hispanicism (*M *= 3.50, *SD *= 0.35). Similar results were obtained for women with a non-history of miscarriage (Americanism (*M *= 2.07, *SD *= 0.80); Hispanicism (*M *= 3.47, *SD *= 0.38). These results reflect a low level of acculturation to the American culture in both groups with no significant differences among them.

### Health-Related characteristics

In this study, 30% of participants reported having a history of miscarriage and 70% did not report a history of miscarriage. As shown in Table 3, Latinas who experienced a miscarriage had significantly more children (*M *= 1.83, *SD *= 1.36), b = .40, *p* < .001 and more pregnancies (*M *= 3.93, *SD *= 1.99), b = .74, *p* < .001 than those who did not report miscarriages (*M *= 1.87, *SD *= 1.69). In addition, Latinas with a history of miscarriages were less likely to rate their health as good or very good (N* *= 66, 74%), b = 11.18, *p* < .001 compared with Latinas with no miscarriage history (*N *= 185, 89%). There were no significant differences in having health insurance or a regular healthcare provider. Further, although there was no significant difference identified for history of STI, the top three STI's reported by participants with a history of miscarriage were candida albicans/bacterial vaginosis (24%), genital herpes/HPV (7%), and chlamydia/urethritis/ drip (4%).

**Table 3:** Health-Related characteristics of latinas with a history of miscarriag*e.*

Characteristics	No Miscarriage (*n* = 207)		Miscarriage (*n* = 89)		*b*	*p*
	*M*	*SD*	*M*	*SD*		
Number of children	1.22	1.13	1.83	1.36	0.40	<.001
Number of pregnancies	1.87	1.69	3.93	1.99	0.74	<.001
	*n*	%	*n*	%	*χ²/b*	*p*
Self-rated Good Health	185	89	66	74	11.18	.001
Has Regular Healthcare Provider	66	32	28	31	0.01	.943
History of STI	73	35	27	30	0.68	.411
**IPV**						
Physically Hurt	8	4	5	6	0.46	.500
Insulted	39	19	17	19	0.00	.958
Threatened	9	4	6	7	0.74	.389
Screamed at	25	12	10	11	0.04	.837

### Intimate partner violence

Forty percent of the participants reported an episode of intimate partner violence. Of them, 10.9% reported being physically hurt, 47.1% reported being insulted, 12.6% reported being threatened, and 29.4% being screamed at. However, there were no significant differences in IPV between Latinas who experienced or did not experience a miscarriage.

## Discussion

This study contributes new data about different characteristics of Latinas who have and have not experienced a miscarriage. In this study, 30% of Latinas experienced at least one miscarriage, which matches the estimated U.S. national rates (30%–40%) of miscarriages (1, 2). In this analysis, Latinas who experienced a miscarriage were significantly older, had more pregnancies, and more children. It is expected that as women age, they may have more pregnancies compared to younger women, thus they might have more children but also, they will have increased risk or likelihood for miscarriage since increased maternal age is a main risk factor for increased genetic malformations to occur (8).

Evidence suggests that higher Latina acculturation is associated to pregnancy complications such as preterm births and gestational hypertension but there is a paucity of research between acculturation and miscarriage (14). In this study, acculturation was not found to be significant between Latinas with and without miscarriage. However, both groups of Latinas scored higher on the Hispanicism dimension indicating a low level of acculturation to the American culture even after having lived in the US for an average of 8 years. In addition to higher Hispanicism, the similar prevalence in miscarriages among Latinas in this study compared to the estimated U.S. national rates, could be related to the “Latina paradox” which is related to the favorable birth outcomes despite social disadvantages, which are associated to cultural, social factors, and the social network that provides protective factors and a behavioral context for healthy births (19, 20). Even when these mechanisms are not completely clear, we know that they are relevant to Latinas who experience several social determinants of health that could decrease their access to health services and screenings to prevent or receive appropriate care if they experience a miscarriage. In this study, women had low income, few had health insurance, and the majority were Spanish speaking which could complicate their access to care when experiencing a miscarriage. Existing literature indicates that poorer patient experiences are reported among those with lower socioeconomic status (21). Future research should explore the role of certified nurse midwives and how they can contribute to reducing these disparities and potentiate protective factors among Latina women as well as on the relationship between acculturation and pregnancy loss among Latinas.

Existing evidence supports the association between intimate partner violence (IPV) and miscarriage in other populations, including minority populations (9, 22). However, in this study, there was no significance found between those with and without history of miscarriage in terms of IPV. This could be because the HITS survey asked about IPV exposure solely during the month prior to study participation and not at other time periods of the participants' life which may have increased the exposure of women to IPV. Furthermore, the prevalence of IPV was high in both groups of Latina women studied. Thus, future studies should assess the relationship between history of IPV and miscarriage and its health-related impacts on larger and varied Latina populations.

Findings from this study indicate that Latinas with history of miscarriage significantly rate their overall health as worse than those without history of miscarriage. There is existing international literature indicating that reproductive history, particularly parity and early vs. late childbearing, influences women's health and their overall self-rated perceptions of health later in life (23–26). However, self-rated health among Latinas has been largely understudied particularly within the realm of miscarriage and pregnancy loss. One study comparing self-rated health among 5,620 White and African American women (another minority group), 50 years old or older, with childbearing history (number of children and pregnancy loss) stated that the African American women consistently reported worse physical and overall health as well as lower education and income than the White women. Furthermore, having six or more children as well as having had a pregnancy loss were both associated with poorer self-reported health for both races with the stronger association being among the African American women (27). Although the current study did not assess this relationship, it would be worthwhile to investigate since self-rated health has consistently predicted future health, functional status, and mortality across cultures and populations (27, 28). Given the scarcity of research regarding self-rated health and pregnancy loss particularly among the Latina population, warrants this type of study.

Nurses and certified nurse midwives have the opportunity to be at the forefront of conducting these needed studies that would help inform the nursing community on how to tailor their care and provide quality patient education that would improve health outcomes for Latinas who have had a miscarriage. Early pregnancy planning, prenatal counseling, and culturally tailored education for Latinas of childbearing age may promote healthy behaviors, decrease risk for miscarriage, and improve overall health status perceptions. The literature shows that patient education is crucial in miscarriage management and should be focused on maternal health, risk factors, preparing for future pregnancy if appropriate, contraception, and mental health among others (3). A caring organizational culture and supportive leadership from certified nurse midwives will ensure early consultation for pregnant Latina women and facilitate care continuity, improving Latina's health outcomes.

### Limitations

We used a cross sectional design and thus cannot establish causal relationships since we did not measure the timing of the miscarriage(s). Data was self-reported by the participants. The analysis was completed with a largely homogenous sample of Latina women, making it challenging to find significant differences between the two groups. Further, most participants (53%) were of Cuban origin which limits generalizability of results to Latinas from other national origins.

### Recommendations

The results of this study demonstrate many dimensions related to Latina miscarriages that require further exploration. Therefore, it will be important that certified nurse midwives who work with Latinas receive appropriate training to help better understand the Latino cultural components related to miscarriage and contribute to providing culturally sensitive care during and after a miscarriage. In addition, efforts need to be made to motivate and recruit more Latinos pursuing careers as nurses, certified nurse midwives, and terminal degrees (e.g., Doctor of Nursing Practice or Doctor of Philosophy in Nursing).

This study also highlights the lack of research on miscarriage among Latinas. Therefore, qualitative and quantitative studies on miscarriages in Latin women are urgently needed to better understand their risk factors and unique needs for post-miscarriage care. In addition, research results will help to better identify Latinas at risk for miscarriage or its adverse-related outcomes and help to develop public health policies that focus on preventing and managing miscarriage among Latinas.

## Conclusion

This study is one of the few that has investigated characteristics of Latinas who have experienced a miscarriage. Certified nurse midwives are encouraged to provide Latinas with culturally tailored education on the importance of early prenatal care for optimal pregnancy outcomes. Certified nurse midwives also have an important role advocating for Latina's access to health and providing support to navigate the system and the decision-making process in cases of miscarriage. Further research is warranted to determine the role of IPV, acculturation, and overall health perceptions among Latinas who experience miscarriage.

## Data Availability

The original contributions presented in the study are included in the article/Supplementary Materials, further inquiries can be directed to the corresponding author.
