# Supplementary material for: Assessment of miscarriage factors among Latinas who live in the U.S.: a cross-sectional study
Source: Front Glob Womens Health. 2023 Apr 26;4:1127695. doi: 10.3389/fgwh.2023.1127695 (PMC10169706; doi:10.3389/fgwh.2023.1127695)
Supplement: Supplementary file 1 [file Table1.docx]

**Appendix**

**Questionnaires**

**(DEM-B) SEPA Demographic Intake Form**

1a. What is your gender?: **○** Man **○** Woman

1b. How old are you? __________ years

1c. Please tell me where you were born (country of birth).

|  | United States |  | Costa Rica |  | Honduras |  |  | Puerto Rico |
| --- | --- | --- | --- | --- | --- | --- | --- | --- |
|  | Argentina |  | Cuba |  | Mexico |  |  | Uruguay |
|  | Bolivia |  | Dominican Republic |  | Nicaragua |  |  | Venezuela |
|  | Brazil |  | Ecuador |  | Panama |  |  | Other |
|  | Chile |  | El Salvador |  | Paraguay |  |  | (Specify |
|  | Colombia |  | Guatemala |  | Peru |  |  | ___________) |

1a. Years living in U.S. __________

2. What is your preferred language? **○**English **○**Spanish

3. What is your current relationship status?

**○**Single **○**In a relationship, not legally married **○**Married

**○**Divorced **○**Separated **○**Widowed

4. Are you currently living with your spouse or partner?

**○**YES **○**NO **○**Not Applicable

5. How many children do you have? __________ **○** None ***(SKIP to 6)***

5a. I am going to ask you some questions about your children

| Age | Do they live with you? | |
| --- | --- | --- |
|  | ○ Yes | ○ No |
|  | ○ Yes | ○ No |
|  | ○ Yes | ○ No |
|  | ○ Yes | ○ No |
|  | ○ Yes | ○ No |

If there are more than 5 children, bubble here: **○**

5b. Do any of your children live in another country? **○**YES **○**NO ***(SKIP to 6)***

5b1. If yes, give their ages, country where they live, and number of years you have lived apart

(in another country) from them. List from youngest to oldest. If there are more than 4 children living in another country, bubble here: **○**

Child 1 Age _____ Country ____________________ Years apart _____

Child 2 Age _____ Country ____________________ Years apart _____

Child 3 Age _____ Country ____________________ Years apart _____

Child 4 Age _____ Country ____________________ Years apart _____

6. What religion are you?

**○**Baptist **○**Jehovah's Witness **○**Presbyterian

**○**Christian **○**Jewish **○**Protestant

**○**Episcopalian **○**Methodist **○**Catholic

**○**Evangelist/Pentecostal **○**Muslim **○**None

**○**Other Christian (Specify ______________________________________)

**○**Other Non - Christian (Specify ______________________________________)

7. How often do you attend religious services? Would you say...

**○**More than once a week **○**Less than once a month

**○**Weekly **○**Only on special days

**○**Monthly (1+) **○**Not at all

8. Do you consider yourself...? ***(INTERVIEWER: Read all choices)***

**○**Not religious **○**Somewhat religious **○**Very religious

9. How strongly do the beliefs of your religion influence your life? ***(INTERVIEWER: Read all choices)***

**○**Not at all **○**Somewhat **○**Very much **○**Not Applicable

10. How many years of education have you completed? __________

11. Are you currently employed? **○**YES **○**NO

12. What is your main occupation/job title in **this** country? ________________________________________

13. Last month, what was your family income? $______________ dollars ***(provide an estimation of your monthly family income in dollars)***

14. How many people this money maintain? __________

15. Do you have health insurance? **○**YES **○**NO

16. How do you usually pay for **your own** health care? ***(Bubble only one)***

**○**Private Insurance Plan (not provided at work) **○**Medicare

**○**Private Insurance Plan (provided at work) **○**Out of Pocket

**○**Medicaid **○**Don't Pay

**○**Other (Specify _________________________________)

17. Where do you usually go when you are sick or want advice about your health? ***(Bubble all that apply)***

| **○** Yes **○** No | Clinic |
| --- | --- |
| **○** Yes **○** No | Nurse Practitioner |
| **○** Yes **○** No | Doctor’s Office |
| **○** Yes **○** No | Emergency Room |
| **○** Yes **○** No | “Curandero” |
| **○** Yes **○** No | Family Member, Friend, or Neighbor |
| **○** Yes **○** No | Other (Specify:____________________________________________) |

18. Do you have a regular doctor or healthcare provider? **○**YES **○**NO

19. How many months have been since your last saw to the doctor or health care provider? ______________ Months ***(enter the response in number of months, if less than 1 month enter the appropriate number: 1 week= 0.25 months, 2 weeks=0.5 months, 3 weeks= 0.75 months).***

20. How many times were **you** in the emergency room in the past three months for **your**

health problems? _____________

21. How would you describe your health in the past three months?

**○**Poor **○**Fair **○**Good **○**Very Good

***(BAS) Bidimensional Acculturation Scale (Marin and Gamba, 1996)***

***INTERVIEWER READ OUT LOUD: I will now ask you about your language preferences***

| **(Bubble only one response to the following**  **questions)** | **Almost never**  **(1)** | **Sometimes**  **(2)** | **Often**  **(3)** | **Almost always**  **(4)** |
| --- | --- | --- | --- | --- |
| 1. How often do you speak English? | **○** | **○** | **○** | **○** |
| 2. How often do you speak in English with your friends? | **○** | **○** | **○** | **○** |
| 3. How often do you think in English? | **○** | **○** | **○** | **○** |
| 4. How often do you speak Spanish? | **○** | **○** | **○** | **○** |
| 5. How often do you speak in Spanish with your friends? | **○** | **○** | **○** | **○** |
| 6. How often do you think in Spanish? | **○** | **○** | **○** | **○** |

|  | **Very poorly**  **(1)** | **Poorly**  **(2)** | **Well**  **(3)** | **Very**  **well**  **(4)** |
| --- | --- | --- | --- | --- |
| 7. How well do you speak English? | **○** | **○** | **○** | **○** |
| 8. How well do you read in English? | **○** | **○** | **○** | **○** |
| 9. How well do you understand television programs in English? | **○** | **○** | **○** | **○** |
| 10. How well do you understand radio programs in English? | **○** | **○** | **○** | **○** |
| 11. How well do you write in English? | **○** | **○** | **○** | **○** |
| 12. How well do you understand music in English? | **○** | **○** | **○** | **○** |
| 13. How well do you speak Spanish? | **○** | **○** | **○** | **○** |
| 14. How well do you read in Spanish? | **○** | **○** | **○** | **○** |
| 15. How well do you understand television programs in Spanish? | **○** | **○** | **○** | **○** |
| 16. How well do you understand radio programs in Spanish? | **○** | **○** | **○** | **○** |
| 17. How well do you write in Spanish? | **○** | **○** | **○** | **○** |
| 18. How well do you understand music in Spanish? | **○** | **○** | **○** | **○** |
|  | **Almost never**  **(1)** | **Sometimes**  **(2)** | **Often**  **(3)** | **Almost always**  **(4)** |
| 19. How often do you watch television programs in English? | **○** | **○** | **○** | **○** |
| 20. How often do you listen to radio programs in English? | **○** | **○** | **○** | **○** |
| 21. How often do you listen to music in English | **○** | **○** | **○** | **○** |
| 22. How often do you watch television programs in Spanish? | **○** | **○** | **○** | **○** |
| 23. How often do you listen to radio programs in Spanish? | **○** | **○** | **○** | **○** |
| 24. How often do you listen to music in Spanish? | **○** | **○** | **○** | **○** |

**(HSH) Health & Sexual History (Peragallo, 1998)**

*INTERVIEWER READ OUT LOUD:* Now we are going to talk about some personal health issues. These might be health issues about things you do or things that might affect you. Please remember that your name does not appear anywhere in this questionnaire and that everything you say is completely private and confidential. This questionnaire will be kept in a locked office where only project management will have access to information. So, please try to answer honestly.

1. How many children do you have? _____

**WOMEN QUESTION (MEN DO NOT ANSWER THIS QUESTION)**

1. How many:
2. times have you been pregnant? _____ **(INTERVIEWER: If 0, skip to Q. 3)**
3. living children? _____
4. elective abortions? _____
5. miscarriages? _____
6. of your children have died? _____

**INTERVIEWER:** Check that (a = b + c + d + e); Be able to explain for any discrepancies that don’t add up - multiple births, etc… ___________________________________________________________________

____________________________________________________________________________________

1. What types of birth control did you or your partner(s) use in **last** **three months**?

**INTERVIEWER: Please read out entire list. (Bubble all that apply)**

| Oral Contraceptives  (“The Pill” or the birth control pill) | ○ Yes ○ No | Rhythm method / calendar | ○ Yes ○ No |
| --- | --- | --- | --- |
| Diaphragm | ○ Yes ○ No | Depo Provera (injections) | ○ Yes ○ No |
| Intra-Urinary Device (IUD) | ○ Yes ○ No | Norplant | ○ Yes ○ No |
| Tubal Ligation (tubes tied) or a Hysterectomy | ○ Yes ○ No | Withdrawal | ○ Yes ○ No |
| Partner had a vasectomy | ○ Yes ○ No | The Patch | ○ Yes ○ No |
| Female condoms (Reality ®) | ○ Yes ○ No | The Day After Pill | ○ Yes ○ No |
| Male condoms | ○ Yes ○ No | NONE / No form of birth control used | ○ Yes ○ No |
| Foams or Jellies or Film | ○ Yes ○ No | Other  (Specify__________________) | ○ Yes ○ No |

1. Why haven’t you or your partner(s) used birth control during the **past three months**?

**INTERVIEWER: Ask only if NONE was answered in question 1 above. (Bubble all that apply)**

| Partner/Woman can’t get pregnant | ○ Yes ○ No | Partner/Woman is pregnant now | ○ Yes ○ No |
| --- | --- | --- | --- |
| Don’t like birth control | ○ Yes ○ No | Supplies unavailable | ○ Yes ○ No |
| Don’t believe in birth control/Religion | ○ Yes ○ No | Steady relationship | ○ Yes ○ No |
| Too expensive | ○ Yes ○ No | Partner does not want ME to use any | ○ Yes ○ No |
| Partner does not want to use any | ○ Yes ○ No | Partner does not like ME to use any | ○ Yes ○ No |
| Partner does not like to use condoms | ○ Yes ○ No | Postmenopausal | ○ Yes ○ No |
| Want to get pregnant | ○ Yes ○ No | Other (Specify ________________) | ○ Yes ○ No |

1. Have you ever been tested for HIV or any other sexually transmitted infections?

○ Yes

○ No **(INTERVIEWER: Skip to Q. 8)**

○ Don’t Know

○ Refused

1. Which sexually transmitted infections have you been **TOLD** you have?

**(INTERVIEWER: Read out the entire list)**

| **(Bubble all that apply)** | **YES** | **NO** | **(Bubble all that apply)** | **YES** | **NO** |
| --- | --- | --- | --- | --- | --- |
| a. Syphilis | ○ | ○ | g. Hepatitis B or C | ○ | ○ |
| b. Genital Herpes/HPV/ | ○ | ○ | h. Gonorrhea/Clap | ○ | ○ |
| c. Vaginal/Genital warts | ○ | ○ | i. Chlamydia/Urethritis/Drip | ○ | ○ |
| d. Pubic lice/Crabs | ○ | ○ | j. Trichomonas | ○ | ○ |
| e. HIV/AIDS | ○ | ○ | k. Pelvic Inflammatory Disease (PID) | ○ | ○ |
| f. Candida Albicans/ Bacterial Vaginosis | ○ | ○ | k. Other  (SPECIFY:_____________) | ○ | ○ |

**WOMEN QUESTIONS (MEN DO NOT ANSWER THIS SECTION)**

1. How long ago did you have your last Pap Smear? _________ months ***(Please enter the number in months, e.g. 2 years you should enter 24 months; if less than 1 month enter the appropriate number: 1 week= 0.25 months, 2 weeks=0.5 months, 3 weeks= 0.75 months). If never had a Pap Smear, enter -88.***
2. How long ago did you have your last mammogram? _________ months ○ Never ○ NA

***(Please enter the number in months, e.g. 2 years you should enter 24 months; if less than 1 month enter the appropriate number: 1 week= 0.25 months, 2 weeks=0.5 months, 3 weeks= 0.75 months). If never had a mammogram, enter -88.***

**(HITS) HITS Tool for Intimate Partner Violence Screening (Sherin, 2003)**

***INTERVIEWER READ OUT LOUD:*** Please indicate the option that best represents the frequency with which you main partner acts in the way depicted **in the past month.**

| How often does your partner? | Never  (1) | Rarely  (2) | Sometimes  (3) | Fairly often  (4) | Frequently  (5) |
| --- | --- | --- | --- | --- | --- |
| 1. Physically hurt you | ○ | ○ | ○ | ○ | ○ |
| 1. Insulted or talked down to you | ○ | ○ | ○ | ○ | ○ |
| 1. Threatened you with harm | ○ | ○ | ○ | ○ | ○ |
| 1. Screamed or cursed at you | ○ | ○ | ○ | ○ | ○ |
